# Why do hospital mastectomy rates vary? Differences in the decision-making experiences of women with breast cancer

**DOI:** 10.1038/bjc.2011.141

**Published:** 2011-05-10

**Authors:** L J M Caldon, K A Collins, D J Wilde, S H Ahmedzai, T W Noble, A Stotter, D M Sibbering, S Holt, M W R Reed

**Affiliations:** 1 Department of Oncology, University of Sheffield, School of Medicine and Biomedical Sciences, Beech Hill Road, Sheffield S10 2RX, UK; 2 Centre for Health and Social Care Research, Sheffield Hallam University, 32 Collegiate Crescent, Sheffield S10 2BP, UK; 3 University Hospitals of Leicester, Glenfield Hospital, Groby Road, Leicester LE3 9QP, UK; 4 Derby Hospitals NHS Foundation Trust, Uttoxeter Road, Derby DE22 3NE, UK; 5 Chesterfield Royal Hospital NHS Foundation Trust, Calow, Chesterfield S44 5BL, UK

**Keywords:** breast cancer, mastectomy, breast conservation surgery, breast conservation therapy, treatment variation, treatment decision making

## Abstract

**Background:**

Hospital mastectomy rates vary. This study explores the relationship between mastectomy rates and breast cancer patients’ consultation and decision-making experiences with specialist clinicians.

**Methods:**

Qualitative semi-structured interviews were conducted with 65 patients from three purposively selected breast units from a single UK region. Patients provided with a choice of breast cancer surgery (breast conservation therapy (BCT) or mastectomy) were purposively recruited from high, medium and low case-mix-adjusted mastectomy rate units.

**Results:**

Low mastectomy rate unit patients’ consultation and decision-making experiences were markedly different to those of the medium and high mastectomy rate breast units. Treatment variation was associated with patients’ perception of the most reassuring and least disruptive treatment; the content and style of information provision (equipoise or directed); level of patient participation in decision making; the time and process of decision making and patient autonomy in decision making. The provision of more comprehensive less directive information and greater autonomy, time and support of independent decision making were associated with a lower uptake of BCT.

**Conclusion:**

Variation in hospital mastectomy rates was associated with differences in the consultation and decision-making experiences of breast cancer patients. Higher mastectomy rates were associated with the facilitation of more informed autonomous patient decision making.

Guidelines state, when breast conservation therapy (BCT) is not contraindicated on clinical grounds, women with breast cancer should be offered a choice between BCT and mastectomy (National Collaborating Centre for Cancer, 2009). Neither treatment is superior in terms of survival (in cancers up to 5 cm diameter) (van Dongen *et al*, 2000; Fisher *et al*, 2002), physical or psychological morbidity (except body image) (Irwig and Bennetts, 1997; McCready *et al*, 2005). However, hospital breast units worldwide demonstrate widely varying practice (Goel *et al*, 1997; Morrow *et al*, 2001; Ishizaki *et al*, 2002; Caldon *et al*, 2005), which is not explained by case mix (Caldon *et al*, 2005).

It is commonly supposed that if women were given more choice over their surgery, the majority would select BCT. Although some evidence supports this (Degner *et al*, 1997; Mastaglia and Kristjanson, 2001), there is also evidence that not all women given an informed choice will elect to undergo BCT (Keating *et al*, 2002; Lantz *et al*, 2005; Caldon *et al*, 2008; Collins *et al*, 2009).

Patients’ health care decisions are subject to many influences. At the time of diagnosis, women with breast cancer hold pre-existing values, concerns and knowledge, which can influence their treatment preferences, including prior information and experience of breast cancer (Collins *et al*, 2009), body image values (Schou *et al*, 2002; Molenaar *et al*, 2004), cancer recurrence fears (Nold *et al*, 2000; Schou *et al*, 2002; Molenaar *et al*, 2004) and attitudes towards radiotherapy (Schou *et al*, 2002; Molenaar *et al*, 2004). Clinicians may also influence patients’ treatment decisions by recommending a specific treatment or communicating a particular preference (Smitt and Heltzel, 1997; Nold *et al*, 2000; Molenaar *et al*, 2004). Limited information is available on how consultations between breast cancer patients and their clinicians influence patients’ treatment choices.

This is the fourth paper published from a research study utilising quantitative and qualitative methodology to examine variation in hospital breast unit mastectomy rates in a UK region. Previous papers confirmed case mix does not explain variation in breast unit mastectomy rates (Caldon *et al*, 2005), specialist breast cancer clinicians possess treatments preferences based on cancer, patient and clinician characteristics (Caldon *et al*, 2007), and patients now desire more autonomy in choosing their breast cancer treatment (Caldon *et al*, 2008). This paper presents the findings of semi-structured interviews conducted among patients clinicians identified as having been given a choice of surgery (BCT or mastectomy) from three breast units demonstrated as having high, medium and low case-mix-adjusted mastectomy rates. It explores the consultation and treatment decision-making experiences of women newly diagnosed with breast cancer to identify themes associated with variation in breast unit mastectomy rates.

## Patients and methods

### Study design

Qualitative methodology was chosen to explore how specialist clinicians influenced patients’ choice of surgery (BCT or mastectomy). Semi-structured interviews were employed to capture rich data on the topics of pre-determined interest, while providing sufficient flexibility to capture emergent themes in allied areas of interest.

The interview schedule was developed by the research team, including experienced qualitative researchers, two surgeons, one breast care nurse (BCN) and two consumer representatives.

Prior to conduct, Multicentre Research Ethics Committee and relevant Local Ethics and Research Governance approvals were obtained.

### Setting and sample

The study was conducted in three purposively selected specialist breast units from a single UK region: Trent with population approximately 5 million. The observed : expected mastectomy rates of the high, medium and low mastectomy rate units were 1.30, 1.03 and 0.48, respectively, based on the case-mix adjustment of cancers <15 mm diameter (*n*=1399) diagnosed through the region's National Health Service Breast Screening Programme (1997–2001) (Caldon *et al*, 2005); that is correcting for patient age, cancer grade and location within the breast, the high mastectomy rate unit performed 30% more mastectomies, the medium rate unit 3% more mastectomies and the low rate unit 52% less mastectomies than expected for this subgroup of cases treated by the region. All units followed similar routine practice guidelines and had similar access to radiotherapy and breast reconstruction.

Patients were recruited via the previously published questionnaire phase of the wider research study from high, medium and low mastectomy rate breast units (Caldon *et al*, 2008). Potential questionnaire participants were purposively identified by their own breast unit clinicians as being those offered a choice between BCT and mastectomy, and able to provide informed consent to participate in research. Patients were approached following their surgery. The recruitment process and eligibility criteria are fully described elsewhere (Caldon *et al*, 2008). To explore the experiences of the range of patients given treatment choices, a sampling frame (Marshall, 1996) was used to identify potential interviewees from questionnaire participants willing to be interviewed, balancing treatment undertaken and breast unit mastectomy rate. Reports of similar studies suggest that response saturation could be achieved by interviewing approximately 20 patients per breast unit. By convention, this is the point where no new themes or information emerge on data analysis (Marshall, 1996). Seventy-seven per cent (274 out of 357) of patients completing the questionnaire agreed to be contacted for follow-up interviews. The first 65 consecutive eligible patients fulfilling the sampling frame requirements were interviewed over an 8-month period. Data analysis was performed alongside data collection and interviews ceased on attainment of response saturation. Twenty patients were interviewed from the low and medium mastectomy rate units, and 25 from the high mastectomy rate unit.

The participants were a median 58 years old (range 33–73 years). The mean time between surgery and interview was 6 weeks (range 1.9–20.6 weeks). Overall, 74% underwent BCT (*n*=48) and 26% mastectomy (*n*=16). Four of the breast conservation patients required additional surgery for more extensive disease than identified pre-operatively: one underwent re-excision of margins from the high mastectomy rate unit and three had mastectomy (two from the medium and one from the low mastectomy rate unit).

### Data

The interview schedule was designed to provide a description of the patient's surgical treatment decision-making experience. Information about consultations with multi-disciplinary breast team members was gathered, focussing on the content and style of consultations, patients’ comprehension of information provided and how these influenced treatment decisions. Information was also sought on patients’ treatment and preconceptions, information and experiences of breast cancer.

Interviews varied in length between 26 and 120 min (median 51 min). All were digitally recorded and transcribed verbatim. Field notes were also kept.

### Data analysis (the framework approach)

Interview transcripts and field notes were analysed using the framework approach (Pope *et al*, 2000; Richie *et al*, 2003). This provides a rigorous, comprehensive, systematic method to manage and analyse large volumes of textural data.

The familiarisation phase and generation of the thematic structure were conducted by three researchers and two consumer representatives. To minimise bias and improve the reliability of the analysis, 20% (*n*=12 out of 65) of the interviews were independently coded and charted by two experienced qualitative researchers (one clinical and one non-clinical) as an ongoing process. Subsequent detailed discussion of such analyses ensured consistency and agreement within interpretation and the development of themes throughout analysis. Framework matrices were explored within and between cases, breast units and themes.

#### ^*^Footnote transcription conventions

Any words appearing between two square [] brackets indicate where notes of clarification have been added by the author(s). Ellipsis points indicate where a quotation has been abridged. Following each quotation, the participant's identification number is reported along with their treatment, age and the type of breast unit treated by. This is followed by the page number(s) identifying the extract within the flow of the interview.

## Results

There was heterogeneity of patients’ experiences within the units. However, the decision-making experiences of women from the medium and high mastectomy rate units were similar, and differences existed between these and those of patients treated by the low mastectomy rate breast unit. The themes identified clustered into two main groups: patient-specific themes and breast unit-specific themes. Table 1 summarises the themes and sub-themes associated with variation in patients’ treatment decisions.

### Patient-specific themes

Concordant with previous studies outlined in the introduction, anecdotal information about patients’ preconceptions regarding breast cancer and its management emerged from the interviews. The patient-specific themes were generally expressed heterogeneously within and across the breast units, and were predominantly independent of breast unit influence. However, two patient-specific sub-themes were associated with variation in patients’ treatment: perception of the most reassuring treatment option and perception of the least disruptive treatment option. Table 2 summarises these sub-themes and factors.

#### Most reassuring treatment option.

Although patients were aware of and accepted the equivalence of survival with BCT and mastectomy, the extensiveness of surgery often influenced perception of safety. Many choosing mastectomy said this option reduced their anxiety about the completeness of cancer excision, the need for further treatment and some felt it improved long-term outcome.

^*^‘[I could] never have…had as much peace of mind if I’d just had the lump removed, …what if they’ve missed a little bit round it.’ [Patient 1, mastectomy, age 42, medium mastectomy rate unit, p5]

Some felt because they were offered BCT, their cancer was not as bad or harmful as it might have been. A few believed that this meant they were more likely to be cured. This was particularly predominant among patients of the low mastectomy rate unit.

‘Total mastectomy…conjures up…you are riddled with cancer…wide local excision…contains your thoughts that it's not as bad as your brain's telling you.’ [Patient 27, BCT, age 61, high mastectomy rate unit, p9]

While most patients based their decisions on information provided by the clinicians, some based it on health beliefs within family or community.

‘Mastectomy…much bigger operation, but feel that the problem's gone, it's not going to recur in the breast tissue because it's not there anymore.’ [Patient 31, mastectomy, age 64, high mastectomy rate unit, p11]

Patients also utilised anecdotal experiences to decide their treatment.

‘I do know several people who’ve had just the lump removed and in a year or two they’ve had to go back and have a mastectomy.’ [Patient 35, mastectomy, age 65, high mastectomy rate unit, p9]

#### Least disruptive treatment option.

Patients also chose their surgery based on what seemed the least disruptive treatment option. For some it meant the option, which would cause least disruption to their wider life and commitments during treatment, with a shorter hospital stay (BCT) or overall treatment (mastectomy).

‘It was a heck of a lot [of a time commitment]…that would absolutely devastate my life…as I live it, so unless…I’d no choice, I didn’t want [radiotherapy].’ [Patient 16, mastectomy, age 73, medium mastectomy rate unit, p9]

For others, the least disruptive treatment option was determined by the potential impact of surgery on body image or sexual relationship. So breast preservation was extremely important and mastectomy would be a reminder of the cancer.

‘If you’re disfigured…it's a constant reminder that you’ve got, had or in remission of cancer…every time you look at yourself.’ [Patient 42, BCT, age 60, high mastectomy rate unit, p23]

Although body image concerns predominated among those choosing BCT and fear of recurrence predominated among those choosing mastectomy, many vacillated, trading between their concerns regarding safety and recurrence, and the disruption of normality and body image.

Although these have been classified as patient-specific themes, the decision-making considerations of patients undergoing BCT from the low mastectomy rate unit were less likely to include the possibility of postoperative re-excision or the need for radiotherapy. In contrast, medium and high mastectomy rate unit patients voiced such information readily and utilised it when making treatment decisions.

### Breast unit-specific themes

The breast unit-specific sub-themes related to treatment variation were information content and style; time and decision-making process and patient autonomy in decision making.

#### Information content and style.

Patients’ treatment choices were influenced by the content and style of information provided by their breast units. While individual variation between surgeons of the same breast unit was noted, more pronounced differences were evident between surgeons of the different breast units. Table 3 summarises these sub-themes and factors.

Low mastectomy rate unit patient accounts focussed on their clinicians’ reassurance regarding their cancer and its treatment, along with treatment recommendations. Patients were more likely to only be offered BCT, even if requesting information about mastectomy or expressing a preference for it.

‘I went in and…[the consultant] said what they wanted to do, this operation and take it away [BCT]…And I just said to her ‘Well why don’t you just take the whole lot off…and she said…she didn’t think there was any need whatsoever to go to those extremes.’ [Patient 60, BCT, age 57, low mastectomy rate unit, p13]

In contrast, patients from the medium and high mastectomy rate units typically recounted much more detailed descriptions of the information provided about both BCT and mastectomy, what undergoing each would involve, the potential consequences and the amount of time they had for decision making.

‘He went through the various options that I could take…having the lump removed and going for follow-up treatment radiotherapy, and which would be a five-week [course]…or the mastectomy. He also went through the pros and cons of each one…and wrote this down.’ [Patient 9, mastectomy, age 50, medium mastectomy rate unit, p4]

Patients described the different roles clinicians had in providing information for making decisions. Doctors were viewed as primary information providers, while BCNs reiterated, reinforced and explored information needs. Patients of the high mastectomy rate unit often described extensive discussions with their BCN and a thorough process of checking understanding. Low mastectomy rate unit patients felt that BCNs were generally uncomfortable extending the consultant's consultation. This was most notable when patients expressed a treatment preference at variance with their doctors’ recommendations.

‘I said, ‘Well what happens if I just have the whole breast off,’ and she [BCN] said, ‘Well that's something you’ll have to discuss with your surgeon, I can’t tell you that.’ [Patient 60, BCT, age 57, low mastectomy rate unit, p7]

Within the information style sub-theme, four factors were associated with patients’ choices, contextualising information, emphasis and minimisation, accessibility of information and treatment recommendations.

*Contextualising information*

Patients being involved in the choice of their surgery were contextualised differently. The medium and high mastectomy rate units tended to introduce the concept and explain the rationale early within the consultation, leading patients to expect involvement in decision making.

‘He was saying…some women like the choice…some…prefer one to the another… they’re equal, there is no better option. The choice is yours.’ [Patient 42, BCT, age 60, high mastectomy rate unit, p5]

Most patients from the low mastectomy rate unit did not describe the provision of such information.

‘He said, ‘I’m sorry, it is malignant, but it is very, very small, you will need to have an operation. I think it would be appropriate for you to have a lumpectomy.’ [Patient 63, BCT, age 59, low mastectomy rate R unit, p7]

When patients received information about BCT and had no recollection of a preliminary introduction of a choice of treatments being available, they tended to assume that it was either the only option available or the recommendation of the clinician or unit.

The other type of contextualisation observed was how individual treatment options were framed; whether this was in an open, directive or dismissive manner.

‘[The surgeon] said, ‘Normally people with one that's as small as you have this incision [BCT], and at the end she mentioned mastectomy, but you got the impression she didn’t think you should go along that line.’ [Patient 64, BCT, age 59, low mastectomy rate unit, p16]

*Emphasis and minimisation*

Clinicians often stressed or minimised certain cancer characteristics (small size and the early nature), which influenced patients’ perceptions of the extent of their disease and which treatment they should undergo.

‘[The surgeon] said it was only a diddy [very small] one, that it hadn’t grown very big and, it had started to invade slightly but they hoped it wasn’t in the lymph nodes…the way [they]…explained the cancer made me feel a mastectomy wasn’t necessary.’ [Patient 52, BCT, age 53, low mastectomy rate unit, p6]

*Accessibility of information*

The language and consultation styles adopted by clinicians influenced the accessibility of information to patients. Language varied from everyday to bio-medical. Clinicians’ consultation styles varied between open, tailored, two-way dialogues to a more prescriptive style.

‘He [the surgeon] accepted the fact that I had a brain, I’d been looking at things and…took my background into account [and]…we talked about things as a couple of adults.’ [Patient 6, BCT, age 56, medium mastectomy rate unit, p7] ‘[The consultant] wasn’t really listening to what I was saying…Rather than it be a discussion between us, I felt it was a one-sided discussion.’ [Patient 62, BCT, age 44, low mastectomy rate unit, p10–11]

*Treatment recommendations*

There was a tendency for low mastectomy rate breast unit clinicians to volunteer a treatment plan early within the consultation, containing recommendations based on what the clinician felt was most appropriate.

‘I think it would be appropriate for you to have a lumpectomy… So he didn’t actually say, ‘Which would you prefer?’ …and he of course was the second person who’d said what the course of action would be.’ [Patient 63, BCT, age 59, low mastectomy rate unit, p7]

In contrast, the medium and high mastectomy rate clinicians tended to provide patients with more open and comprehensive information and the opportunity to choose.

‘They were very clear that this was going to be my choice and that they wouldn’t push one against the other. They just simply presented all the facts about the two.’ [Patient 33, BCT, age 57, high mastectomy rate unit, p6]

Some patients, immediately after diagnosis, felt underprepared for a role in decision making and asked their clinician's recommendation. At this point, the medium and high mastectomy rate clinicians usually spent more time discussing options and emphasising the time and support available for such a decision, while low mastectomy rate clinicians tended to recommend a treatment.

The force of clinicians’ recommendations varied in a spectrum between encouragement to consider both options with a gentle steer, to a form of recommendation where patients felt they were denied their preferred treatment.

‘I was, ‘Get it off, cut it off.’ And she was…very kind, very understanding but…very gently steering me to the outcome that she wanted.’ [Patient 48, BCT, age 38, low mastectomy rate unit, p16] ‘Mr __ said to me…‘I don’t like doing mastectomies’ …So…there was no discussion really on having my whole breast off…And every time I brought up the subject…he wasn’t really listening to what I was saying.’ [Patient 62, BCT, age 44, low mastectomy rate unit, p12]

#### Time and process of decision making.

The process of decision making varied between the breast units. It was generally more rapid in the low mastectomy rate unit and patients reported pressure to make an immediate decision. This was reinforced in the low mastectomy rate unit by all, but one consultant frequently consenting patients on the day of diagnosis, immediately after treatment discussion.

‘I found it a very hard decision [I]…couldn’t decide at all…I’d got to decide there and then whether I had a mastectomy or a lumpectomy…so I decided on the lumpectomy and within minutes all the paperwork was there.’ [Patient 51, BCT, age 51, low mastectomy rate unit, p7]

Although most recalled being told they could alter their consent should they change their mind, in practice, this could prove a challenge.

‘…after thinking long and hard…I rang up the breast care nurses the week before my operation was due, and I made an appointment to go and speak to Mr__…because I’d decided by then I wanted the whole thing off. …So I went to see him …and this is quite important to me really – I felt I was talked out of the mastectomy…there was no sort of discussion…he wasn’t really listening…I feel that I…went with what he said, rather than what [I wanted]…I’d gone in there to tell him I wanted a mastectomy and to rip up the consent form and sign another one. …I’d done the consent form…but I was told all the way through that…that can be ripped up half an hour before the operation.’ [Patient 62, BCT, age 44, low mastectomy rate unit, p9–10]

In contrast, the medium and high mastectomy rate unit's patients found the process of decision making less rushed. They were almost universally provided with clear information early in the consultation about the date of their surgery and were told they had until then to make a decision. Patients often expressed that they needed to spend time away from the clinical environment following their diagnosis, to think and reach a decision.

‘Knowing I’d got this fortnight…And being told two or three times I didn’t have to make a decision there and then, I could leave it right ‘til the morning of the operation…helped. So…I didn’t panic…I’d got this [time], I was going to come home, and there's no place like home for thinking things through without any pressure.’ [Patient 31, mastectomy, age 64, high mastectomy rate unit, p8]

#### Autonomy: level of patient participation in decision making.

A large proportion of patients initially assumed that they would be given a treatment plan, and expressed surprise when offered choice of treatment instead. However, after an explanation about why a choice was offered, most desired participation in the decision.

Breast units had a substantial impact on the autonomy patients assumed in the selection of their treatment. Discrepancies in power arose following patients’ new diagnosis of cancer. Disparities in the knowledge and experience of patients and clinicians were compounded by the amount of time patients perceived they had for decision making. Discrepancies were more pronounced among low mastectomy rate breast unit patients and were reinforced or moderated by clinicians’ information provision and consultation skills.

‘[The consultant's] not intimidating in that way…but you do feel as though you’re slightly walking on eggshells…the trouble is you’re in a very, very vulnerable position…there are some questions you wouldn’t ask because you don’t want to upset people, because you feel they’re in charge of your life.’ [Patient 49, mastectomy, age 59, low mastectomy rate unit, p17–18]

Doctors seemed to exert the more powerful influence, and BCNs often adopted empowering techniques.

‘Because there was two doctors in the room and I didn’t have a [Breast Care] nurse in with me…when I had a breast care nurse with me before, we were all involved in the conversation so I felt I had my support with me…I just felt a little bit, not intimidated, because he's not an intimidating man, I just.’ [Patient 62, BCT, age 44, low mastectomy rate unit, p11–12]

Patients of the low mastectomy rate unit tended to experience simpler and more rapid decision making than patients from the other units, as their surgery tended to be directed by clinicians. In contrast, decision making within the medium and high mastectomy rate units tended to be more complex, with patients often describing periods of reflection and deliberation. Although some found the decision-making experience quite challenging, on reflection, patients often felt proud of the achievement, owned the decision and expressed confidence in the treatment chosen.

‘I’ve changed my mind as things have progressed…initially I was angry…I thought they should make the choice, they’re the experts. But now I’m glad that I had the choice because I’ve made the choice and I’ve got to live with it. And I am quite sure that I made the right choice.’ [Patient 24, mastectomy, age 60, high mastectomy rate unit, p27–28]

## Discussion

This study provides new information on the clinical consultation and decision-making experiences of women with breast cancer who are suitable for BCT, and may explain the reasons why mastectomy rates vary between different hospital breast units. While patient-specific themes affected patients’ treatment choices, the specialist clinicians’ and units’ influence was often the foremost. In particular, the paper elucidates how patients’ decisions were influenced by clinicians in both overt and subtle ways. Differences in patients’ information, as well as the experience of information provision and decision making were associated with variation in mastectomy rates.

The study reveals how patients’ decisions were affected by the treatment options they are offered, the content and style of the information provided by their clinicians (equipoise or directed) and the level of patient autonomy and time provided for decision making. While individual variation between surgeons of the same breast unit was noted, more pronounced differences were evident between surgeons of the different breast units. Among patients participating in the study, those from the low mastectomy rate unit recalled less comprehensive more directive information, less autonomy and less time for decision making. Conversely, patients from the high and medium mastectomy rate units described the provision of more comprehensive less directive information, together with greater support and time for more autonomous decision making. The themes associated with differences in breast unit mastectomy rates are summarised in Table 4. The provision of treatment choices is appropriate given patients’ preference for increasing autonomy in decision making (Mastaglia and Kristjanson, 2001; Janz *et al*, 2004; Lantz *et al*, 2005; Caldon *et al*, 2008) and the potential benefits of involving patients in health care decisions, including optimising patient well being (Stewart, 1995; Street and Voigt, 1997; Deadman *et al*, 2001; Hack *et al*, 2006) and satisfaction with the decision-making experience as well as treatment (Street and Voigt, 1997; Keating *et al*, 2002; Lam *et al*, 2003; Lantz *et al*, 2005; Hack *et al*, 2006). However, evidence from the current study (conducted among patients whom clinicians believed they had offered treatment choices) and others demonstrate patients’ acquiescence to professionals’ preferences and recommendations (Smitt and Heltzel, 1997; Nold *et al*, 2000; Molenaar *et al*, 2004).

Clinicians’ directedness could represent an attempt to counteract patient misconceptions. If this were the case, it is reasonable to anticipate that this group would be better informed. However, in this study, the converse seemed to be the case; with high and medium mastectomy rate unit patients often displaying more comprehensive knowledge of treatment including BCT, than their low mastectomy rate unit counterparts.

Clinicians are often unsuccessful at gauging their patients’ preferences (Bruera *et al*, 2002; Janz *et al*, 2004), and empowering patients require reciprocal change in clinicians (Butow *et al*, 2004) if any, but the most active of decision makers are to truly choose their treatment. These findings may encourage clinicians to adopt a more open, tailored and empowering approach in consultations and foster an active role in treatment selection among those patients who desire it (Mastaglia and Kristjanson, 2001; Janz *et al*, 2004; Lantz *et al*, 2005; Caldon *et al*, 2008). Methods to empower patient decision making might include communication skills training for clinicians, developing the BCN's role and the adoption of decision support interventions (O’Connor *et al*, 2003; Gysels and Higginson, 2007).

One of the findings of the study is that many patients need to be given time to recover from the shock of their diagnosis of cancer, before they are able to engage in decision making. If patients’ more autonomous decision making is to be supported, the provision of directive information and treatment recommendations should be deferred and carefully targeted. This should mean that those who will benefit from participating in decision making (Street and Voigt, 1997; Keating *et al*, 2002; Lam *et al*, 2003; Janz *et al*, 2004; Lantz *et al*, 2005; Hack *et al*, 2006) get the opportunity to do so. It should also permit clinicians to identify and direct the minority who definitively do not want a role in choosing their treatment, so that the provision of recommendations is more likely to be based on the patients’ preference.

Although decision-making experiences varied, most patients were satisfied with their surgical treatment and decision-making experience. The exceptions were active decision makers from the low mastectomy rate breast unit whose treatment preference differed from the treatment they were recommended, and passive decision makers from the medium and high mastectomy rate units who wanted more direction. If the trend for patients desiring increasing autonomy in decision making (Mastaglia and Kristjanson, 2001; Janz *et al*, 2004; Lantz *et al*, 2005; Caldon *et al*, 2008) continues, units adopting a more prescriptive approach may find their patients’ satisfaction diminished.

A potential limitation of the study is that participants were interviewed following the completion of the decision-making experience. The authors were limited to approaching patients after surgery due to the sensitive nature of exploring such experiences in a vulnerable group of patients and ethical constraints within the United Kingdom. The interviews were conducted within a similar timeframe to many other qualitative studies exploring treatment decision making. Although it is possible that recall bias and *post hoc* justification may have influenced the findings, we do not believe that *post hoc* justification is likely to be a major influence. Participants clearly articulated their experiences and concerns, including where conflict existed between their preferences and outcomes. The findings are also concordant with the results of Collins *et al's* (2009) questionnaire-based study, which was conducted in the United States throughout breast cancer patients’ decision-making pathway.

This study was conducted within a UK region with demonstrable variation in the surgical treatment of breast cancer (Caldon *et al*, 2005). The authors identified high, medium and low mastectomy rate breast units with adjustment of raw mastectomy rate data for the units’ case mix, and recruited representative units to participate. Compared with other UK breast units, the low mastectomy rate unit reflects outlying practice, whereas the high mastectomy rate unit lies within the medium rate spectrum (BCCOM, 2006). Some UK units have mastectomy rates approaching 80%, and it is possible that such units have similar practices to that of the low mastectomy rate unit in our study, but with a preference or lower threshold for directing patients towards mastectomy.

The results of this and other studies (Keating *et al*, 2002; Lantz *et al*, 2005; Caldon *et al*, 2008; Collins *et al*, 2009) appear to defy the conventional assumption that high BCT rates arise from a more fully informed group of patients being permitted to choose their own treatment. The findings instead suggest that improving patient knowledge and involvement in decision making may result in a reduction in the uptake of BCT.

Although this study was conducted among patients with breast cancer, the issue of understanding treatment variation and involving patients in health care decisions is becoming a national and international priority. Our findings may help to explain and address practice variation in this and other oncology specialities where treatment options exist, such as de-functioning ileostomy and reversal rates in colorectal cancer (Koperna, 2003) and the management of localised prostate cancer (Zeliadt *et al*, 2006).

## Figures and Tables

**Table 1:** Themes and sub-themes associated with variation in patients’ treatment decisions

*Patient-specific themes*
Most reassuring treatment option
Least disruptive treatment option

*Breast unit-specific themes*
Information content and style
Time and process of decision making
Autonomy: level of patient participation in decision making

**Table 2:** Patient-specific sub-themes associated with variation in patients’ treatment decisions

*Most reassuring option*
*Safety and fears*
Cancer fully removed
Survival
Local recurrence
Minimise the psycho-physical impact of the cancer diagnosis (implication of better prognosis with less extensive surgery)

*Anecdotal information/experiences*
Positive or negative anecdotal experiences

*Least disruptive option*
*Minimise impact on life, relationships and social commitments*
Social commitments
Family (especially partners and dependents)
Minimise hospital treatment experience
BCT – shorter in-patent stay
Mastectomy – shorter overall cancer treatment (minimise need for radiotherapy and re-excision)

*Minimise the psycho-physical impact of surgery*
Body image disruption minimised with BCT
Potential impact on partners and relationships
Mastectomy as a constant reminder of cancer

Abbreviation: BCT=breast conservation therapy.

**Table 3:** Information content and style sub-themes

*Information content*
*The facts*
Options
Treatment details
Potential consequences
Comparison of treatments
Time for decision making

*Information style: spectrum from equipoise to forceful direction*
*Contextualising information*
Framing of involvement in decision making
Framing of the options: open/directive/dismissive

*Emphasis and minimisation*
Cancer size
Implication of ‘early cancer’
Mastectomy as more a more extreme option

*Accessibility*
Language and terminology: everyday language *vs* bio-medical
Consultation style: two-way dialogue *vs* prescriptive; tailored *vs* universal

*Recommendations*
Provided or not
Overt *vs* perceived
Volunteered *vs* provided on request
Timing of recommendation: early *vs* delayed

**Table 4:** Summary of themes associated with breast unit treatment variation

**Low mastectomy rate unit**	**Medium and high mastectomy rate units**
Less comprehensive information	More comprehensive information
	
*Active direction of choice*	*Reluctance to direct choice*
More directive information	Less directive information
More volunteering of clinician recommendations	Less volunteering of clinician recommendations
Less active support of autonomous patient decision making	Active support of autonomous patient decision making
Time pressure for decision making	Lack of time pressure for decision making
	
*Process factors*	*Process factors*
Consent early: at diagnosis or 1 week after diagnosis	Consent later: at pre-assessment clinic or pre-operatively

## References

[bib1] BCCOM (2006) Breast Cancer Clinical Outcome Measures (BCCOM) Project: analysis of the management of symptomatic breast cancers diagnosed in 2002. 1-3-2006. http://www.wmpho.org.uk/wmciu/documents/BCCOM_annual_report.pdf Ref Type: Report

[bib2] Bruera E, Willey JS, Palmer JL, Rosales M (2002) Treatment decisions for breast carcinoma: patient preferences and physician perceptions. Cancer 94: 2076–2080 11932912 10.1002/cncr.10393

[bib3] Butow P, Devine R, Boyer M, Pendlebury S, Jackson M, Tattersall MH (2004) Cancer consultation preparation package: changing patients but not physicians is not enough. J Clin Oncol 22: 4401–4409 15514382 10.1200/JCO.2004.66.155

[bib4] Caldon LJ, Walters SJ, Ratcliffe J, Reed MW (2007) What influences clinicians’ operative preferences for women with breast cancer? An application of the discrete choice experiment. Eur J Cancer 43: 1662–1669 17555955 10.1016/j.ejca.2007.04.021

[bib5] Caldon LJ, Walters SJ, Reed JA, Murphy A, Worley A, Reed MW (2005) Case-mix fails to explain variation in mastectomy rates: management of screen-detected breast cancer in a UK region 1997–2003. Br J Cancer 92: 55–59 15611797 10.1038/sj.bjc.6602264 PMC2361751

[bib6] Caldon LJ, Walters SJ, Reed MW (2008) Changing trends in the decision-making preferences of women with early breast cancer. Br J Surg 95: 312–318 17853508 10.1002/bjs.5964 PMC11440001

[bib7] Collins ED, Moore CP, Clay KF, Kearing SA, O’Connor AM, Llewellyn-Thomas HA, Barth Jr RJ, Sepucha KR (2009) Can women with early-stage breast cancer make an informed decision for mastectomy? J Clin Oncol 27: 519–525 19114703 10.1200/JCO.2008.16.6215

[bib8] Deadman JM, Leinster SJ, Owens RG, Dewey ME, Slade PD (2001) Taking responsibility for cancer treatment. Soc Sci Med 53: 669–677 11478545 10.1016/s0277-9536(00)00369-5

[bib9] Degner LF, Kristjanson LJ, Bowman D, Sloan JA, Carriere KC, O’Neil J, Bilodeau B, Watson P, Mueller B (1997) Information needs and decisional preferences in women with breast cancer. JAMA 277: 1485–1492 9145723

[bib10] Fisher B, Anderson S, Bryant J, Margolese RG, Deutsch M, Fisher ER, Jeong JH, Wolmark N (2002) Twenty-year follow-up of a randomized trial comparing total mastectomy, lumpectomy, and lumpectomy plus irradiation for the treatment of invasive breast cancer. N Engl J Med 347: 1233–1241 12393820 10.1056/NEJMoa022152

[bib11] Goel V, Olivotto I, Hislop TG, Sawka C, Coldman A, Holowaty EJ (1997) Patterns of initial management of node-negative breast cancer in two Canadian provinces. British Columbia/Ontario Working Group. CMAJ 156: 25–35 9006561 PMC1226853

[bib12] Gysels M, Higginson IJ (2007) Interactive technologies and videotapes for patient education in cancer care: systematic review and meta-analysis of randomised trials. Support Care Cancer 15: 7–20 17024500 10.1007/s00520-006-0112-z

[bib13] Hack TF, Degner LF, Watson P, Sinha L (2006) Do patients benefit from participating in medical decision making? Longitudinal follow-up of women with breast cancer. Psychooncology 15: 9–19 15669023 10.1002/pon.907

[bib14] Irwig L, Bennetts A (1997) Quality of life after breast conservation or mastectomy: a systematic review. Aust N Z J Surg 67: 750–754 9396988 10.1111/j.1445-2197.1997.tb04573.x

[bib15] Ishizaki T, Imanaka Y, Hirose M, Kuwabara K, Ogawa T, Harada Y (2002) A first look at variations in use of breast conserving surgery at five teaching hospitals in Japan. Int J Qual Health Care 14: 411–418 12389807 10.1093/intqhc/14.5.411

[bib16] Janz NK, Wren PA, Copeland LA, Lowery JC, Goldfarb SL, Wilkins EG (2004) Patient-physician concordance: preferences, perceptions, and factors influencing the breast cancer surgical decision. J Clin Oncol 22: 3091–3098 15284259 10.1200/JCO.2004.09.069

[bib17] Keating NL, Guadagnoli E, Landrum MB, Borbas C, Weeks JC (2002) Treatment decision making in early-stage breast cancer: should surgeons match patients’ desired level of involvement? J Clin Oncol 20: 1473–1479 11896094 10.1200/JCO.2002.20.6.1473

[bib18] Koperna T (2003) Cost-effectiveness of defunctioning stomas in low anterior resections for rectal cancer: a call for benchmarking. Arch Surg 138: 1334–1338 14662534 10.1001/archsurg.138.12.1334

[bib19] Lam W, Fielding R, Chan M, Chow L, Ho E (2003) Participation and satisfaction with surgical treatment decision-making in breast cancer among Chinese women. Breast Cancer Res Treat 80: 171–180 12908820 10.1023/A:1024568732213

[bib20] Lantz PM, Janz NK, Fagerlin A, Schwartz K, Liu L, Lakhani I, Salem B, Katz SJ (2005) Satisfaction with surgery outcomes and the decision process in a population-based sample of women with breast cancer. Health Serv Res 40: 745–767 15960689 10.1111/j.1475-6773.2005.00383.x PMC1361166

[bib21] Marshall MN (1996) Sampling for qualitative research. Fam Pract 13: 522–525 9023528 10.1093/fampra/13.6.522

[bib22] Mastaglia B, Kristjanson LJ (2001) Factors influencing women's decisions for choice of surgery for Stage I and Stage II breast cancer in Western Australia. J Adv Nurs 35: 836–847 11555031 10.1046/j.1365-2648.2001.01921.x

[bib23] McCready D, Holloway C, Shelley W, Down N, Robinson P, Sinclair S, Mirsky D (2005) Surgical management of early stage invasive breast cancer: a practice guideline. Can J Surg 48: 185–194 16013621 PMC3211547

[bib24] Molenaar S, Oort F, Sprangers M, Rutgers E, Luiten E, Mulder J, de Haes H (2004) Predictors of patients’ choices for breast-conserving therapy or mastectomy: a prospective study. Br J Cancer 90: 2123–2130 15150557 10.1038/sj.bjc.6601835 PMC2409497

[bib25] Morrow M, White J, Moughan J, Owen J, Pajack T, Sylvester J, Wilson JF, Winchester D (2001) Factors predicting the use of breast-conserving therapy in stage I and II breast carcinoma. J Clin Oncol 19: 2254–2262 11304779 10.1200/JCO.2001.19.8.2254

[bib26] National Collaborating Centre for Cancer. Early and Locally Advanced Breast Cancer: Diagnosis and Treatment 2009. National Collaborating Centre for Cancer: Cardiff, Wales 20704053

[bib27] Nold RJ, Beamer RL, Helmer SD, McBoyle MF (2000) Factors influencing a woman's choice to undergo breast-conserving surgery versus modified radical mastectomy. Am J Surg 180: 413–418 11182389 10.1016/s0002-9610(00)00501-8

[bib28] O’Connor AM, Stacey D, Entwistle V, Llewellyn-Thomas H, Rovner D, Holmes-Rovner M, Tait V, Tetroe J, Fiset V, Barry M, Jones J (2003) Decision aids for people facing health treatment or screening decisions. Cochrane Database Syst Rev CD001431 10.1002/14651858.CD001431 11686990

[bib29] Pope C, Ziebland S, Mays N (2000) Qualitative research in health care. Analysing qualitative data. BMJ 320: 114–116 10625273 10.1136/bmj.320.7227.114 PMC1117368

[bib30] Richie J, Spencer L, O’Connor W (2003) Carrying out qualitative analysis. In: Richie J, Lewis J (eds) Qualitative Research Practice. Sage Publications: London, pp 219–262

[bib31] Schou I, Ekeberg O, Ruland CM, Karesen R (2002) Do women newly diagnosed with breast cancer and consulting surgeon assess decision-making equally? Breast 11: 434–441 14965708 10.1054/brst.2002.0454

[bib32] Smitt MC, Heltzel M (1997) Women's use of resources in decision-making for early-stage breast cancer: results of a community-based survey. Ann Surg Oncol 4: 564–569 9367022 10.1007/BF02305537

[bib33] Stewart MA (1995) Effective physician-patient communication and health outcomes: a review. CMAJ 152: 1423–1433 7728691 PMC1337906

[bib34] Street Jr RL, Voigt B (1997) Patient participation in deciding breast cancer treatment and subsequent quality of life. Med Decis Making 17: 298–306 9219190 10.1177/0272989X9701700306

[bib35] van Dongen JA, Voogd AC, Fentiman IS, Legrand C, Sylvester RJ, Tong D, van der SE, Helle PA, van Zijl K, Bartelink H (2000) Long-term results of a randomized trial comparing breast-conserving therapy with mastectomy: European Organization for Research and Treatment of Cancer 10801 trial. J Natl Cancer Inst 92: 1143–1150 10904087 10.1093/jnci/92.14.1143

[bib36] Zeliadt SB, Ramsey SD, Penson DF, Hall IJ, Ekwueme DU, Stroud L, Lee JW (2006) Why do men choose one treatment over another?: a review of patient decision making for localized prostate cancer. Cancer 106: 1865–1874 16568450 10.1002/cncr.21822

